# 1390. Talaromycosis: Differences between Patients with The Anti-interferon-γ Autoantibody and People Living with HIV/AIDS (PLWHA)

**DOI:** 10.1093/ofid/ofad500.1227

**Published:** 2023-11-27

**Authors:** Kawisara Krasaewes, Narootchai Patanadamrongchai, Quanhathai Kaewpoowat, Saowaluck Yasri, Antika Wongthanee, Romanee Chaiwarith

**Affiliations:** Faculty of Medicine, Chiang Mai University, Chiang Mai, Chiang Mai, Thailand; Chiang Mai University, Muang, Phitsanulok, Thailand; Faculty of Medicine, Chiang Mai University, Thailand, Iowa City, Iowa; Faculty of Medicine, Chiang Mai University, Thailand, Iowa City, Iowa; Chiang Mai University, Muang, Phitsanulok, Thailand; Faculty of Medicine, Chiang Mai University, Chiang Mai, Chiang Mai, Thailand

## Abstract

**Background:**

Talaromycosis has been increasingly reported among HIV-uninfected immunocompromised conditions in an endemic area. This study aimed to compare characteristics, and mortality associated with talaromycosis with adult-onset immunodeficiency caused by the anti-interferon-gamma autoantibody (anti-IFN- γ AAb) to those of people living with HIV/AIDS (PLWHA).

**Methods:**

A retrospective cohort study was conducted at Maharaj Nakorn Chiang Mai Hospital, Thailand in adults with confirmed documentation of HIV infection or anti-interferon-gamma autoantibody, with a diagnosis of talaromycosis.

**Results:**

Thirty-two patients with anti-IFN- γ AAbs and 64 PLWHA were enrolled. Anti-IFN- γ AAbs patients were older, more likely to have nodules and abscess skin lesions, bone and joint infections, leukocytosis, abnormal chest radiograph, and less likely to have fungemia. Time from the first symptom to treatment was longer in patients with anti-IFN- γ AAbs (44.5 days VS. 28.0 days, p-value=0.006). The 24-week-mortality rate was 9.4% (3 patients) in patients with anti-IFN- γ AAbs and 18.8% (12 patients) in PLWHA (p-value=0.225).

**Conclusion:**

The clinical features of talaromycosis in patients with anti-IFN-γ AAbs were different from PLWHA. Clinicians in areas endemic for talaromycosis should be aware of differing characteristics of talaromycosis in anti-IFN- γ AAbs patients.

**Disclosures:**

**All Authors**: No reported disclosures

